# Supplementary material for: Bias assessment of a test-negative design study of COVID-19 vaccine effectiveness used in national policymaking
Source: Nat Commun. 2023 Jul 6;14:3984. doi: 10.1038/s41467-023-39674-0 (PMC10325974; doi:10.1038/s41467-023-39674-0)
Supplement: Supplementary file 1 — Supplementary Information [file 41467_2023_39674_MOESM1_ESM.pdf]

Supplementary figure titles and legends

Supplementary Figure 1. Key pathways under investigation in the current study. It should be noted that not all possible pathways are represented in the below figure, however, the key pathways are represented for exposure misclassification, outcome misclassification, confounding, deferral bias, riskier behaviour after vaccination and vaccination itself associated with COVID-19.

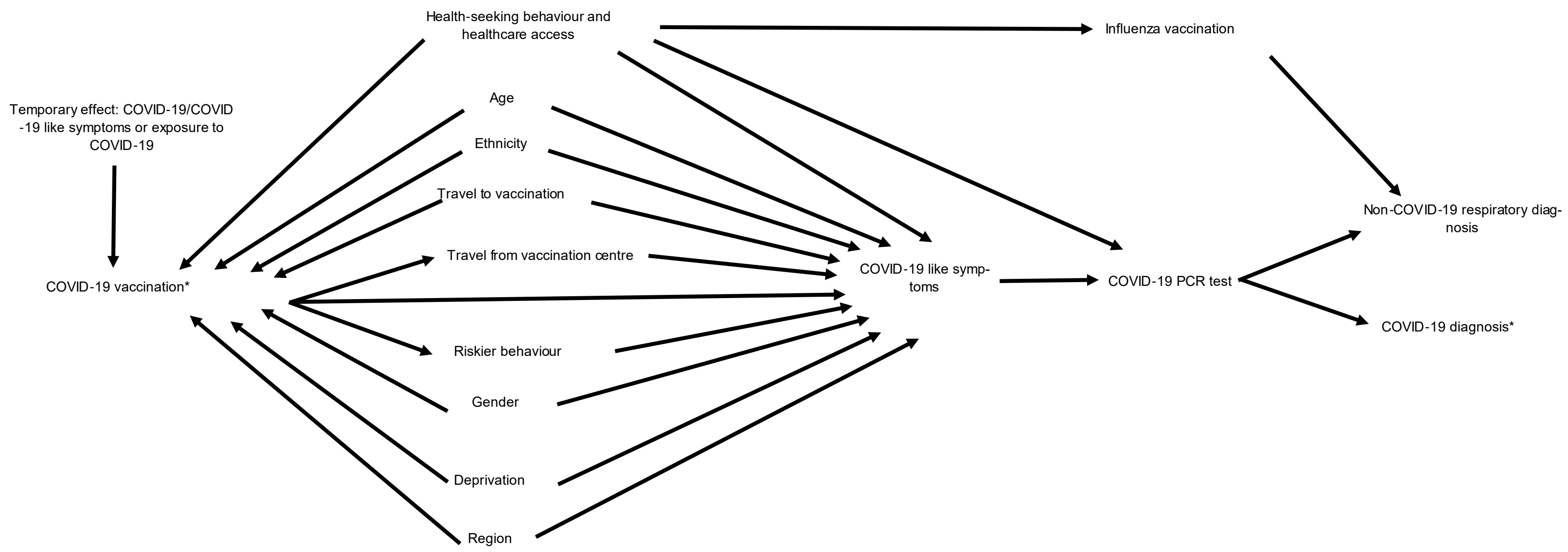

Abbreviations: PCR: polymerase chain reaction. Note: * represents classified as exposed or diagnosed.

Supplementary Figure 2. Histograms representing difference in days between NIMS and questionnaire vaccination date for both A) dose 1 and B) dose 2. ***Negative values indicate that the self-reported vaccination date is earlier than NIMS.***

A)

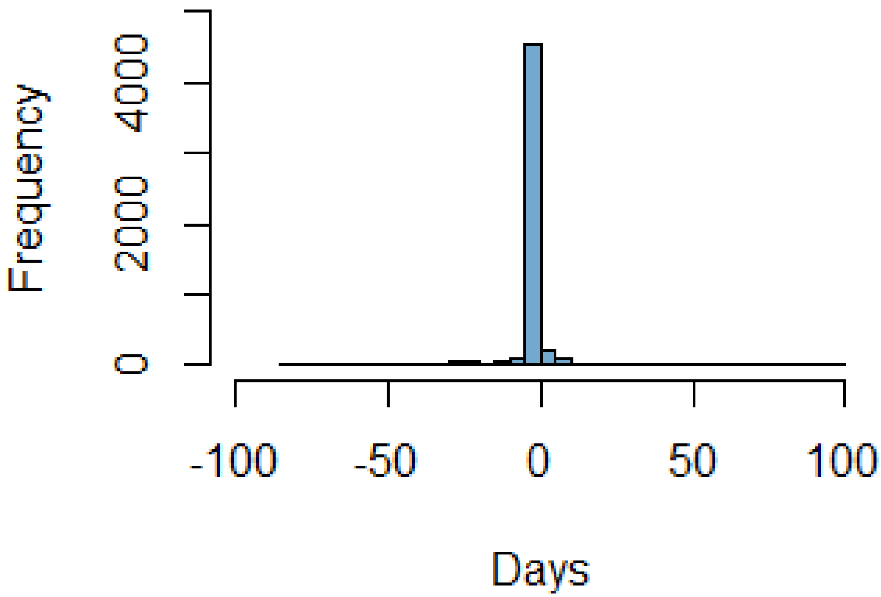

B)

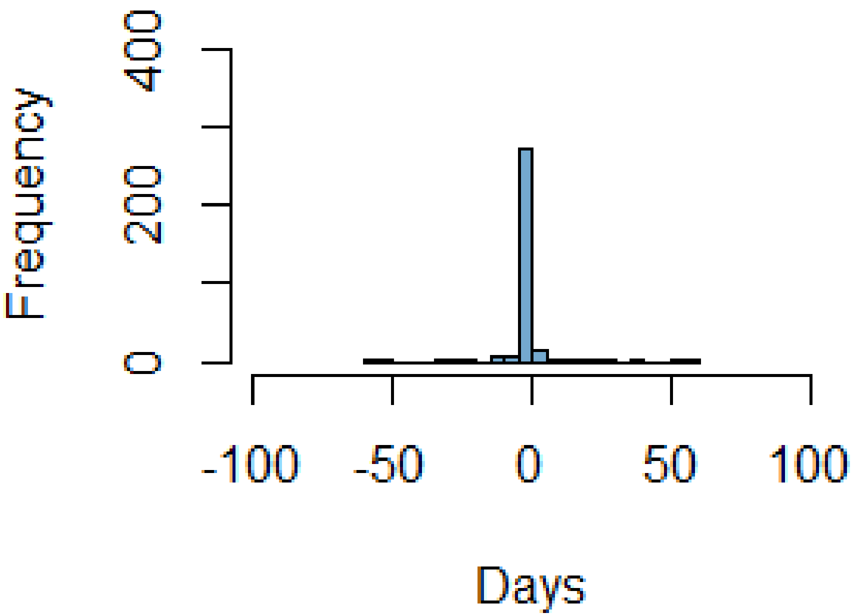

Supplementary Figure 3. Histogram representing difference in days between SGSS and questionnaire onset date (assuming questionnaire is the earlier date). ***Negative values indicate that the self-reported onset date is earlier than SGSS.***

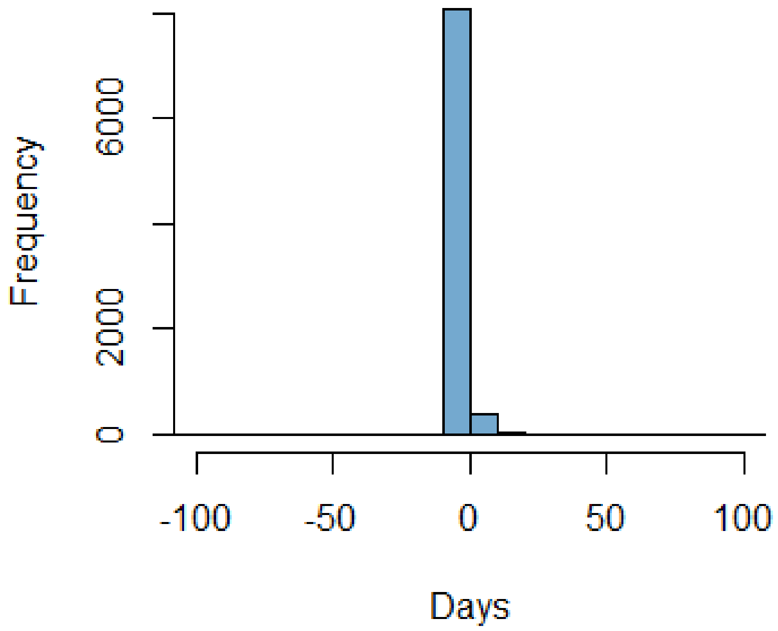

Supplementary table titles and legends

Supplementary Table 1. Summary of Responses in the Questionnaire

**Responses to the questionnaire summarised**				
		**n**	**N**	**%**
Respondents:		8,648	23,713	36.5%
Self-reported vaccination status at date of questionnaire response:	Vaccinated:	8,518	8,613	98.9%
	Non-vaccinated:	95	8,613	1.2%
Self-reported CEV status*:		2,337	8,648	27.1%
Self-reported comorbidities:	Chronic heart disease:	663	8,648	7.7%
	Chronic kidney disease:	158	8,648	1.8%
	Chronic liver disease:	29	8,648	0.3%
	Chronic respiratory disease:	881	8,648	10.2%
	Asthma requiring medication:	1,032	8,648	11.9%
	Cancer:	486	8,648	5.6%
	Organ or bone transplant:	18	8,648	0.2%
	HIV/immunodeficiency:	12	8,648	0.14%
	Immunosuppression due to medication:	181	8,648	2.1%
	Seizure disorder:	63	8,648	0.7%
	Chronic neurological disease:	112	8,648	1.3%
	Asplenia or dysfunction of the spleen:	22	8,648	0.3%
	BMI ≥40 kg/m^2:	101	8,648	1.2%
Self-reported symptomatic status when requesting PCR COVID-19 test:		5,539	8,459	65.5%
Amongst those with symptoms (N=5,539), health services accessed during illness:	GP:	1,922	5,539	34.7%
	NHS 111:	659	5,539	11.9%
	Hospital:	503	5,539	9.1%
	Emergency department:	216	5,539	3.9%
	Other healthcare:	121	5,539	2.2%
Amongst vaccinated (N=8,518) length of time from invitation to first dose vaccination:	Less than 2 weeks:	6,131	8,518	72.0%
	2-3 weeks:	1,110	8,518	13.0%
	4 or more weeks:	794	8,518	9.3%
	I had my vaccine before I was eligible:	216	8,518	2.5%
	Missing:	267	8,518	3.1%
Amongst vaccinated (N=8,518), mixing patterns after first dose:	Mixed same amount	5,371	8,518	63.1%
	Mixed more:	445	8,518	5.2%
	Mixed less:	2,435	8,518	28.6%
	Missing	267	8,518	3.1%
Amongst those that delayed their vaccination 2-3 weeks (N=1,110), reason for delay	Not aware I was eligible:	182	1,110	16.4%
	No appointments available:	430	1,110	38.7%
	Prefer to wait to be vaccinated:	107	1,110	9.6%
	Delayed because I had COVID-19 or symptoms:	51	1,110	4.6%

	I was isolating:	41	1,110	3.7%
	I did not have time:	8	1,110	0.7%
	Other:	195	1,110	17.6%
	Missing:	96	1,110	8.6%
Amongst those that delayed their vaccination ≥4 weeks (N=794), reason for delay:	Not aware I was eligible:	84	794	10.6%
	No appointments available:	176	794	22.2%
	Prefer to wait to be vaccinated:	73	794	9.2%
	Delayed because I had COVID-19 or symptoms:	201	794	25.3%
	I was isolating:	35	794	4.4%
	I did not have time:	<5	794	-
	Other:	189	794	23.8%
	Missing:	<5	794	-
Amongst vaccinated with two doses (N=6,952), mixing patterns after second dose	Mixed same amount	4,153	6,952	59.7%
	Mixed more:	1,087	6,952	15.6%
	Mixed less:	1,505	6,952	21.6%
	Missing	207	6,952	3.0%
Amongst non-vaccinated (N=95), reason for no vaccination:	Not called for a vaccine:	<5	95	-
	Not aware eligible:	0	95	0.0%
	No appointments available:	<5	95	-
	Prefer to wait to be vaccinated:	32	95	33.7%
	Expect to get vaccinated soon:	5	95	5.3%
	Have been unwell or have had COVID-19:	26	95	27.4%
	I have been isolating:	<5	95	-
	I did not have time:	0	95	0.0%
	Other:	17	95	17.9%
	Missing:	7	95	7.4%

Abbreviations: CEV: clinically extremely vulnerable; n: numerator; N: denominator; PCR: polymerase chain reaction.

*Phrased in the questionnaire as: “Have you been advised you are part of the clinically extremely vulnerable group?”.

Note: since surveys were sent out in March 2021 and individuals were responding to the questionnaire until August 2021. Numbers above reflect self-reported numbers at the time of survey response, rather than at the time of symptom onset in the TNCC study.

Note: Cells <5 have been suppressed and secondary suppression has also been conducted in order to protect patient privacy.

Supplementary Table 2. Baseline characteristics of respondents versus non-respondents using variables from the original study data (NIMS and SGSS)

**Characteristic**	**Respondents, N = 8,648**	**Non-respondents, N = 15,062**	**Percentage absolute difference (respondents – non-respondents)**	**p-value**
**Vaccine status at symptom onset, n (%)**				<0.001
Not vaccinated	1,907 (22.1%)	3,826 (25.4%)	-3.30%	
Vaccinated	6,741 (77.9%)	11,236 (74.6%)	3.30%	
**Test result, n (%)**				<0.001
Negative	6,541 (75.6%)	12,756 (84.7%)	-9.10%	
Positive	2,107 (24.4%)	2,306 (15.3%)	9.10%	
**Age group in years, n (%)**				<0.001
70-74	4,423 (51.1%)	6,561 (43.6%)	7.50%	
75-79	2,335 (27.0%)	3,896 (25.9%)	1.10%	
80-84	1,088 (12.6%)	2,260 (15.0%)	-2.40%	
85-89	516 (6.0%)	1,427 (9.5%)	-3.50%	
=>90	286 (3.3%)	918 (6.1%)	-2.80%	
**Gender, n (%)**				<0.001
Female	4,830 (55.9%)	8,884 (59.0%)	-3.10%	
Male	3,818 (44.1%)	6,178 (41.0%)	3.10%	
**Ethnicity, n (%)**				<0.001
White	8,022 (92.8%)	12,773 (84.8%)	8.00%	
Non-white	308 (3.6%)	1,572 (10.4%)	-6.80%	
Prefer not to say	318 (3.7%)	717 (4.8%)	-1.10%	
**Geographical region, n (%)**				<0.001
East of England	1,060 (12.3%)	1,665 (11.1%)	1.20%	
London	718 (8.3%)	1,738 (11.5%)	-3.20%	
Midlands	1,775 (20.5%)	3,299 (21.9%)	-1.40%	
Northeast and Yorkshire	1,360 (15.7%)	2,278 (15.1%)	0.60%	
Northwest	1,226 (14.2%)	2,221 (14.7%)	-0.50%	
Southeast	1,510 (17.5%)	2,352 (15.6%)	1.90%	
Southwest	999 (12.3%)	1,509 (10.0%)	2.30%	
**IMD quintile, n (%)**				<0.001
1 (most deprived)	1,038 (12.0%)	2,879 (19.1%)	-7.10%	
2	1,337 (15.5%)	2,918 (19.4%)	-3.90%	

3	1,824 (21.1%)	3,091 (20.5%)	0.60%	
4	2,099 (24.3%)	3,196 (21.2%)	3.10%	
5 (least deprived)	2,345 (27.1%)	2,966 (19.7%)	7.40%	
Missing	5	12		
**Week of symptom onset, n (%)**				0.099
January week 1	12 (0.1%)	21 (0.1%)	0.00%	
January week 2	39 (0.5%)	97 (0.6%)	-0.10%	
January week 3	147 (1.7%)	284 (1.9%)	-0.20%	
January week 4	1,724 (19.9%)	2,797 (18.6%)	1.30%	
February week 1	3,004 (34.7%)	5,294 (35.1%)	-0.40%	
February week 2	2,380 (27.5%)	4,207 (27.9%)	-0.40%	
February week 3	1,342 (15.5%)	2,362 (15.7%)	-0.20%	
**Week of COVID-19 test, n (%)**				0.821
February week 1	3,551 (41.1%)	6,211 (41.2%)	-0.10%	
February week 2	2,400 (27.8%)	4,212 (28.0%)	-0.20%	
February week 3	2,697 (31.2%)	4,639 (30.8%)	0.40%	
**Care home status, n (%)**				<0.001
Not care home	8,592 (99.4%)	14,504 (96.3%)	3.10%	
Care home†	56 (0.6%)	558 (3.7%)	-3.10%	
**CEV, n (%)**				<0.001
Not CEV	7,455 (86.2%)	12,311 (81.7%)	4.50%	
CEV	1,193 (13.8%)	2,751 (18.3%)	-4.50%	

Abbreviations: CEV: clinically extremely vulnerable; IQR: interquartile range; IMD: index of multiple deprivation; n: numerator; N = denominator; NIMS: National Immunisation Management System; SGSS: Second Generation Surveillance System.

†Care home status is likely low in the current study because the study only included those tested in the community (pillar 2), individuals tested in care homes or in hospital are usually tested under pillar 1. In addition, care home status was identified in the current study using an algorithm based on address and the list of official care home residencies in the UK, however, some individuals might have been missed through this.

Note: all tests were conducted using Chi squared test.

Supplementary Table 3. Adjusted odds of COVID-19 after two doses of BNT162b2 or one dose of ChAdOx1 amongst questionnaire sample, respondents and non-respondents, by days since vaccination

	Questionnaire sample	Respondents	Non-respondents
	**aOR* (95% CI)**	**aOR* (95% CI)**	**aOR* (95% CI)**
ChAdOx1 1 dose 0-13	0.87 (0.79-0.96)	0.84 (0.72-0.97)	0.81 (0.71-0.93)
ChAdOx1 1 dose 14+	0.74 (0.65-0.85)	0.73 (0.59-0.90)	0.66 (0.55-0.78)
BNT162b2 1 dose 0-13	0.97 (0.87-1.09)	0.90 (0.76-1.07)	0.91 (0.78-1.06)
BNT162b2 1 dose 14+	0.53 (0.47-0.60)	0.47 (0.39-0.58)	0.51 (0.43-0.59)
BNT162b2 2 dose	0.14 (0.09-0.21)	0.12 (0.06-0.21)	0.13 (0.07-0.21)

Abbreviations: aOR: adjusted odds ratio; CI: confidence interval.

*Adjusted for age, gender, ethnicity, geography, index of multiple deprivation, care home status and week of onset.

Supplementary Table 4. Bias or alternative causal pathways, description, analysis, results, limitations and conclusions

**Bias name**	**Definition**	**Analysis**	**Results**	**Limitation**	**Conclusions**
Exposure (vaccination status) misclassification	Occurs when vaccination status is misclassified. In the context of the current study it was thought that exposure misclassification could be introduced through inaccurate vaccination dates in NIMS.	Vaccination dates (first and second) were compared in NIMS and the questionnaire by reporting the number and proportion of individuals that had an earlier vaccination date in NIMS, earlier vaccination dates in the questionnaire and the same date in both. The percentage of self-reported vaccine dates that were within 3 days +/- of NIMS date was reported. Vaccine effectiveness estimates were re-run using self-reported vaccination dates.	There was no evidence of inaccurate vaccination dates in NIMS (first dose: 9.5% of individuals reported a date that was later, and 7.3% reported a date that was earlier in the questionnaire when compared with NIMS). 89.8% of first dose and 93.3% of second dose self-reported vaccination dates were within 3 days +/- of NIMS date. Vaccine effectiveness after two doses of BNT162b2 decreased from 88% (95% CI: 79-94%) to 84% (95% CI: 74-92%).	A number of individuals did not provide their vaccinations dates in the questionnaire. For example, 38.4% of individuals that reported they received their second vaccination in the questionnaire did not provide a vaccination date. Therefore, the comparison of vaccination dates had to be made amongst those with non-missing data and it had to be assumed that those with missing and non-missing dates did not differ in reporting a vaccination date that differed to the date in NIMS. The use of vaccination cards during the COVID-19 pandemic could have reduced the impact of recall bias on self-reported vaccination dates.	Limited evidence of exposure misclassification.
Outcome misclassification	Occurs when outcome status is misclassified. In the context of the current study it was thought that outcome	Identify the proportion of individuals in the questionnaire reporting they were symptomatic. Note: all individuals included in the	65.5% of individuals reported they were symptomatic in the questionnaire, which was lower in vaccinated (64.7%; versus non-vaccinated: 67.4%) and negative	It is not possible to determine whether either or both of these biases are influencing these results. Individuals were only asked to report symptom onset date if different from the date reported in SGSS	Unclear for outcome misclassification from symptomatic status as

	misclassification could be introduced if individuals were incorrectly reporting their symptomatic status or symptom onset date when requesting their PCR test.	original TNCC study were identified as symptomatic in SGSS. This was reported overall and by vaccination and case status. Compared symptom onset dates in SGSS and questionnaire. Vaccine effectiveness estimates were re-run separately excluding those reporting they were asymptomatic and using self-reported symptom onset dates.	controls (59.7%; versus cases: 83.5%). Vaccine effectiveness for two doses of BNT162b2 increased from 88% (95% CI: 79-94%) in respondents of the questionnaire to 92% (95% CI: 84-97%). Symptom onset dates were not too dissimilar in the questionnaire (5.9% of individuals reported an earlier date, whereas, 2.2% of individuals reported a later date in the questionnaire when compared with SGSS). Vaccine effectiveness after two doses of BNT162b2 decreased from 88% (95% CI: 79-94%) to 87% (95% CI: 77-93%).	and therefore individuals that could not remember their onset date potentially could have left this question blank, which would have been incorrectly interpreted as the same date, rather than missing data.	difference by case status could be subject to recall bias (i.e., those that received a positive test were more likely to recall symptoms) or outcome misclassification (i.e., individuals incorrectly reporting they had symptoms in order to access free testing). No or limited evidence of outcome misclassification from COVID-19 symptom onset date.

Vaccinee bias from confounders	Occurs when vaccinated individuals differ systematically from non-vaccinated due to factors such as underlying health, health-seeking behaviour and access to healthcare which are risk factors for protection against the vaccine preventable disease. In the context of the current study, it was thought that confounding was introduced since comorbidities and other COVID-19 risk factors could not be identified in NIMS or SGSS.	Adjusted for risk factors from questionnaire separately in logistic regression models (also adjusting for each of the variables adjusted for in the original TNCC study). Since there were no variables that changed the vaccine effectiveness estimates when adjusted for separately in the model, it was decided that household size, household type and CEV would be adjusted for all together in a *post hoc analysis* since these variables were considered to be of clinical importance.	Adjusting for household size, household type and CEV (i.e., variables thought to potentially be confounders) as well as other variables adjusted for in the original TNCC study decreased the vaccine effectiveness from 88% (95% CI: 79-94%) to 87% (95% CI: 78-93%) after a second dose of BNT162b2.	Relied on accurate reporting of COVID-19 ‘at-risk’ conditions and other COVID-19 risk factors that was not differential by exposure or case status. There are also likely to be other COVID-19 risk factors such as mobility or frailty that could not be measured in the context of the current study.	No or limited evidence of confounding.

Healthy vaccinee bias from vaccine delay when unwell	Occurs when those that receive a vaccination are more likely to be healthy in the short time period around their vaccination, since individuals are asked to defer vaccination if they are unwell or have the vaccine preventable disease. The impact of this can persist if there are inaccuracies with the vaccine preventable disease onset date.	Identified the proportion of individuals that delayed their first vaccination 2+ weeks from their invitation that reported they delayed their vaccines due to COVID-19/COVID-19 symptoms. Identified the proportion of individuals that have not been vaccinated because they were unwell or had COVID-19 infection. Vaccine effectiveness estimates were re-run excluding those reporting they delayed their vaccination because of COVID-19/COVID-19 like symptoms.	Several individuals who delayed their vaccination 4+ weeks (9.3 %) reported they did so because of COVID-19/COVID-19 symptoms (25.3%). Over a quarter (27.4%) of individuals that had not been vaccinated reported they had done so because they had been unwell or because they had COVID-19. Vaccine effectiveness after two doses of BNT162b2 decreased from 88% (95% CI: 79-94%) to 81% (95% CI: 67-90%).		There was some evidence of individuals delaying their vaccinations because they were unwell or because they had COVID-19. However, this had limited effect on vaccine effectiveness estimates.
Riskier behaviour after vaccination	Occurs when individuals might adopt riskier behaviours after they have received a vaccination, which	Identified the proportion of individuals reporting they mixed more after their COVID-19 vaccination (first and second). Then, the odds of COVID-19 amongst those	Individuals did not report mixing more after vaccinations (first dose: 5.2%; second dose: 15.6%). Those that reported they mixed more after their first vaccination dose did not have an increased odds of COVID-19	There was insufficient data to assess the odds of COVID-19 in those that had riskier behaviour after a second vaccination dose. The responses on the questionnaire might have been subject to desirability bias. Since the study	No or limited evidence of riskier behaviour after vaccination.

	increases their risk of infection compared to non-vaccine recipients (**Figure S1**).	that mixed more versus same/less was compared using logistic regression adjusting for age, gender, ethnicity, CEV, immunosuppressive conditions and month of vaccination dose.	(OR: 0.92, 95% CI: 0.68-1.24) compared to those that mixed the same.	population selected for those that only had their first ever COVID-19 test in February 2021, and the population were also those that responded to a governmental survey, it could be that the study population were those with less risky behaviours that the overall English population. The questions were also answered when there were COVID-19 restrictions in the UK and when the prevalence of COVID-19 was high and therefore individuals might have had less risky behaviours for reasons other than their vaccination.	
Vaccination itself associated with higher risk of COVID-19	Occurs when individuals contract the vaccine preventable disease when they are travelling to, from, or even at, their vaccination centre (**Figure S1**).	Identified the mode of transport taken to vaccination centres (first and second dose) stratified by those that had a positive PCR test within 2 weeks (inclusive) of vaccination and then those that had after 2 weeks. Then the odds of COVID-19 within 2 weeks since first vaccination amongst those	Individuals with a positive test within 2 weeks of first dose were more likely to have taken public transport (4.5% vs. 3.5%) but were less likely to have taken car with individuals outside of their household (11.8% vs. 13.9%) compared with those with a positive test more than 2 weeks after vaccination. There was no association with riskier transport to vaccination centre and odds of COVID-19 (car	This was only assessed by the mode of transport that was taken to the vaccination center. There are other potential factors that could have increased an individual's risk, such as the number of individuals queuing at the vaccination center and the mode of transport taken from the vaccination center (if different from the mode taken there).	No or limited evidence of vaccination itself being associated with COVID-19.

		that took riskier modes of transport (car with those outside of household or public transport) to their vaccination centre would be compared to those that took less risky forms of transport (car alone or with members within household or walked/cycled) using a logistic regression adjusting for age, gender, ethnicity, region and IMD.	with members outside household: OR: 1.28, 95% CI: 0.98-1.67; public transport: OR: 1.26, 95% CI: 0.81-2.03) compared to those that walked/cycled/car alone or with members from own household.		

Abbreviations: CEV: clinically extremely vulnerable; CI: confidence interval; IMD: Index of Multiple Deprivation; NIMS: National Immunisation Management System; PCR: polymerase chain reaction; SGSS: Second Generation Surveillance System; TNCC: test-negative-case-control study; VE: vaccine effectiveness.

Supplementary Table 5. Description of key confounders in those with increased or decreased number of doses using self-reported vaccination date onset date (SGSS) versus unchanged vaccination status.

Characteristic	Questionnaire increases number of doses, N = 81	Unchanged, N = 8,377	Percentage point difference (increased doses – unchanged)	p-value (increased doses vs unchanged)	Questionnaire decreases number of doses, N = 189	Percentage point difference (decreased doses – unchanged)	p-value (decreased doses vs unchanged)
**Age**				0.309			0.079
70-74	35 (43.2%)	4,307 (51.4%)	-8.2%		81 (42.9%)	-8.5%	
75-79	29 (35.8%)	2,246 (26.8%)	9.0%		60 (31.7%)	4.9%	
80-84	10 (12.3%)	1,054 (12.6%)	-0.3%		24 (12.7%)	0.1%	
85-89	<5	495 (5.9%)			18 (9.5%)	3.6%	
=>90	<5	275 (3.3%)			6 (3.2%)	-0.1%	
**Gender**				0.805			0.012
Female	47 (58.0%)	4,694 (56.0%)	2.0%		88 (46.6%)	-9.4%	
Male	34 (42.0%)	3,683 (44.0%)	-2.0%		101 (53.4%)	9.4%	
**Ethnicity**				0.528			0.811
White	77 (95.1%)	7,771 (92.8%)	2.3%		173 (91.5%)	-1.3%	
Non-White	<5	299 (3.6%)	-		8 (4.2%)	0.6%	
Prefer not to say	<5	307 (3.7%)	-		8 (4.2%)	0.5%	
**Geographical region**				0.643			0.569
London	6 (7.4%)	695 (8.3%)	-0.9%		17 (9.0%)	0.7%	
South England ex-London	30 (37.0%)	3,468 (41.4%)	-4.4%		71 (37.6%)	-3.8%	
North England	45 (55.6%)	4,214 (50.3%)	5.3%		101 (53.4%)	3.1%	
**IMD**				0.299			0.469
1 (least deprived)	6 (7.4%)	1,007 (12.0%)	-4.6%		25 (13.2%)	1.2%	
2	14 (17.3%)	1,295 (15.5%)	1.8%		28 (14.8%)	-0.7%	
3	13 (16.0%)	1,765 (21.1%)	-5.1%		46 (24.3%)	3.2%	
4	19 (23.5%)	2,044 (24.4%)	-0.9%		36 (19.0%)	-5.4%	
5 (most deprived)	29 (35.8%)	2,261 (27.0%)	8.8%		54 (28.6%)	1.6%	

Missing	0	5			0		
**Week COVID-19 symptom onset**				0.566			0.032
Jan week 1	<5	12 (0.1%)			<5		
Jan week 2	0 (0.0%)	38 (0.5%)	-0.5%		<5		
Jan week 3	<5	146 (1.7%)			0 (0.0%)	-1.7%	
Jan week 4	13 (16.0%)	1,678 (20.0%)	-4.0%		33 (17.5%)	-2.5%	
Feb week 1	36 (44.4%)	2,898 (34.6%)	9.8%		69 (36.5%)	1.9%	
Feb week 2	17 (21.0%)	2,295 (27.4%)	-6.4%		68 (36.0%)	8.6%	
Feb week 3	14 (17.3%)	1,310 (15.6%)	1.7%		18 (9.5%)	-6.1%	
**Week COVID-19 test**				0.630			0.123
Feb week 1	37 (45.7%)	3,442 (41.1%)	4.6%		71 (37.6%)	-3.5%	
Feb week 2	19 (23.5%)	2,316 (27.6%)	-4.1%		65 (34.4%)	6.8%	
Feb week 3	25 (30.9%)	2,619 (31.3%)	-0.4%		53 (28.0%)	-3.3%	
**Care home status**				1			1
Not care home	-	8,323 (99.4%)			-		
Care home	<5	54 (0.6%)			<5		
**CEV NIMS**				0.834			0.609
Not CEV	71 (87.7%)	7,223 (86.2%)	1.5%		160 (84.7%)	-1.5%	
CEV	10 (12.3%)	1,154 (13.8%)	-1.5%		29 (15.3%)	1.5%	

Abbreviations: CEV: clinically extremely vulnerable; IQR: interquartile range; IMD: index of multiple deprivation; n: numerator; N: denominator; NIMS: National Immunisation Management System; SGSS: Second Generation Surveillance System.

Note: all tests were conducted using two-sided Chi squared test.

Note: cells <5 have been suppressed and secondary suppression has also been conducted in order to protect patient privacy.

Supplementary Table 6. Description of key confounders in those self-reporting they were symptomatic versus asymptomatic in the questionnaire.

Characteristic	Symptomatic, N = 5,539	Asymptomatic, N = 2,920	Percentage point difference (symptomatic – asymptomatic)	p-value
**Age**				<0.001
70-74	2,976 (53.7%)	1,376 (47.1%)	6.60%	
75-79	1,492 (26.9%)	783 (26.8%)	0.10%	
80-84	605 (10.9%)	446 (15.3%)	-4.40%	
85-89	301 (5.4%)	201 (6.9%)	-1.50%	
=>90	165 (3.0%)	114 (3.9%)	-0.90%	
**Gender**				<0.001
Female	3,250 (58.7%)	1,468 (50.3%)	8.40%	
Male	2,289 (41.3%)	1,452 (49.7%)	-8.40%	
**Ethnicity**				0.7944
White	5,145 (92.9%)	2,706 (92.7%)	0.20%	
Non-White	193 (3.5%)	110 (3.8%)	-0.30%	
Prefer not to say	201 (3.6%)	104 (3.6%)	0.00%	
**Geographical region**				0.385
London	455 (8.2%)	250 (8.6%)	-0.40%	
South England ex-London	2,273 (41.0%)	1,234 (42.3%)	-1.30%	
North England	2,811 (50.7%)	1,436 (49.2%)	1.50%	
**IMD**				0.875
1 (least deprived)	-	-	-0.10%	
2	849 (15.3%)	467 (16.0%)	-0.70%	
3	1,158 (20.9%)	623 (21.4%)	-0.50%	
4	1,372 (24.8%)	690 (23.6%)	1.20%	
5 (most deprived)	1,501 (27.1%)	790 (27.1%)	0.00%	
Missing	<5	<5		
**Week COVID-19 symptom onset**				0.543
Jan week 1	6 (0.1%)	6 (0.2%)	-0.10%	
Jan week 2	25 (0.5%)	14 (0.5%)	0.00%	

Jan week 3	91 (1.6%)	51 (1.7%)	-0.10%	
Jan week 4	1,092 (19.7%)	596 (20.4%)	-0.70%	
Feb week 1	1,944 (35.1%)	989 (33.9%)	1.20%	
Feb week 2	1,504 (27.2%)	830 (28.4%)	-1.20%	
Feb week 3	877 (15.8%)	434 (14.9%)	0.90%	
**Week COVID-19 test**				0.473
Feb week 1	2,247 (40.6%)	1,221 (41.8%)	-1.20%	
Feb week 2	1,544 (27.9%)	810 (27.7%)	0.20%	
Feb week 3	1,748 (31.6%)	889 (30.4%)	1.20%	
**Care home status**				0.268
Not care home	5,508 (99.4%)	2,897 (99.2%)	0.20%	
Care home	31 (0.6%)	23 (0.8%)	-0.20%	
**CEV NIMS**				<0.001
Not CEV	4,845 (87.5%)	2,449 (83.9%)	3.60%	
CEV	694 (12.5%)	471 (16.1%)	-3.60%	

Abbreviations: CEV: clinically extremely vulnerable; IQR: interquartile range; IMD: index of multiple deprivation; n: numerator; N: denominator; NIMS: National Immunisation Management System; SGSS: Second Generation Surveillance System.

Note: all tests were conducted using two-sided Chi squared test.

Note: cells <5 have been suppressed and secondary suppression has also been conducted in order to protect patient privacy.

Supplementary Table 7. Description of key confounders by those in those with different versus same symptomatic status using self-reported symptomatic date from the questionnaire.

Characteristic	Different onset date, N = 708	Same onset date, N = 7,937	Percentage point difference (different – same)	p-value
**Age**				<0.001
70-74	390 (55.1%)	4,032 (50.8%)	4.3%	
75-79	211 (29.8%)	2,122 (26.7%)	3.1%	
80-84	63 (8.9%)	1,025 (12.9%)	-4.0%	
85-89	32 (4.5%)	484 (6.1%)	-1.6%	
=>90	12 (1.7%)	274 (3.5%)	-1.8%	
**Gender**				0.931
Female	397 (56.1%)	4,431 (55.8%)	0.3%	
Male	311 (43.9%)	3,506 (44.2%)	-0.3%	
**Ethnicity**				0.761
White	653 (92.2%)	7,367 (92.8%)	-0.6%	
Non-White	27 (3.8%)	280 (3.5%)	0.3%	
Prefer not to say	28 (4.0%)	290 (3.7%)	0.3%	
**Geographical region**				0.644
London	65 (9.2%)	652 (8.2%)	1.0%	
South England ex-London	286 (40.4%)	3,283 (41.4%)	-1.0%	
North England	357 (50.4%)	4,002 (50.4%)	0.0%	
**IMD**				0.498
1 (least deprived)	100 (14.1%)	937 (11.8%)	2.3%	
2	100 (14.1%)	1,235 (15.6%)	-1.5%	
3	142 (20.1%)	1,682 (21.2%)	-1.1%	
4	173 (24.4%)	1,926 (24.3%)	0.1%	
5 (most deprived)	193 (27.3%)	2,152 (27.1%)	0.2%	
Missing	0	5		
**Week COVID-19 symptom onset**				0.525

Jan week 1	0 (0.0%)	12 (0.2%)	-0.2%	
Jan week 2	5 (0.7%)	34 (0.4%)	0.3%	
Jan week 3	16 (2.3%)	131 (1.7%)	0.6%	
Jan week 4	147 (20.8%)	1,577 (19.9%)	0.9%	
Feb week 1	235 (33.2%)	2,767 (34.9%)	-1.7%	
Feb week 2	188 (26.6%)	2,191 (27.6%)	-1.0%	
Feb week 3	117 (16.5%)	1,225 (15.4%)	1.1%	
**Week COVID-19 test**				0.761
Feb week 1	300 (42.4%)	3,250 (40.9%)	1.5%	
Feb week 2	192 (27.1%)	2,206 (27.8%)	-0.7%	
Feb week 3	216 (30.5%)	2,481 (31.3%)	-0.8%	
**Care home status**				0.595
Not care home	-	7,884 (99.3%)	-	
Care home	<5	53 (0.7%)	-	
**CEV NIMS**				0.019
Not CEV	631 (89.1%)	6,822 (86.0%)	3.1%	
CEV	77 (10.9%)	1,115 (14.0%)	-3.1%	

Abbreviations: CEV: clinically extremely vulnerable; IQR: interquartile range; IMD: index of multiple deprivation; n: numerator; N: denominator; NIMS: National Immunisation Management System; SGSS: Second Generation Surveillance System.

Note: all tests were conducted using two-sided Chi squared test.

Note: cells <5 have been suppressed and secondary suppression has also been conducted in order to protect patient privacy.

Supplementary materials

Supplementary Materials 1. Questionnaire sent out to individuals aged ≥70 years with a PCR test from February 1 to 21 2021

COVID-19 Vaccine Effectiveness Survey

This is a request for your help from Public Health England about COVID-19. A few weeks ago you had a test for COVID-19 on <<testdate>>. To understand better important questions about how different activities may affect the chance of catching COVID-19 we are asking you if you can help by filling in this short questionnaire. We need you to do this whether your result was positive or negative.

This one-off survey has 4 parts and should take 15-20 minutes to complete.

Part 1: Details about you at the time you had your COVID-19 test

Part 2: COVID-19 Vaccination Details

Part 3: Details about your illness before getting tested for COVID-19

Part 4: Details in the week before you had symptoms (or a test taken if you did not have symptoms)

Please complete this survey as full as possible by Friday 21 May 2021

If the person this was addressed to can't complete the form themselves it would be really helpful if someone could complete this on their behalf if they are happy for you to do so. Please then answer the questions as if you are that person.

Please enter today's date D D / M M / Y Y

Part 1: Details about you at the time you had your COVID-19 test

1. Please enter your forename ______________________

2. Please enter your surname ______________________

3. Please enter your date of D D / M M / Y Y birth

4. What type of accommodation did you live in?

☐ Private home ☐ Care home / Nursing home

☐ Sheltered accommodation ☐ Other

If other please describe

____

5. You were tested for COVID-19 on <<testdate>>. How many people were you living with on <<testdate>>?

☐ 0 ☐ 1 ☐ 2 ☐ 3 ☐ 4 ☐ 5 or more

6. Have you been advised you are part of the clinically extremely vulnerable group?

For example, have you received a letter or telephone call from your GP to say you are at risk and eligible for a vaccine?

☐ Yes ☐ No

7. Do you have any of the following conditions? *Please tick all that apply*

☐ Chronic Heart Disease ☐ Diarrhoea

☐ Chronic Kidney Disease ☐ Chronic Liver Disease

☐ Chronic Respiratory Disease (excluding asthma) ☐ Asthma requiring medication

☐ Cancer ☐ Organ or Bone Marrow Transplant

☐ HIV/Immunodeficiency ☐ Immunosuppression due to medication*

☐ Seizure Disorder ☐ Chronic Neurological Disease

☐ Asplenia or dysfunction of the spleen ☐ BMI $\geq$40 kg/m^2

☐ None of the above

☐ *If you have immunosuppression, please give further details:

____

__

Part 2: COVID-19 Vaccination Details

8. Did you receive an invitation for a COVID-19 vaccine (e.g. from your GP or the NHS)?

☐ Yes

☐ No

9. As of today, have you received one or both doses of the COVID-19 vaccine?

☐ Yes, I received 1 dose

☐ Yes, I received 2 doses

☐ No, I have not had a COVID-19 vaccine *(please go to question 20)*

10. How long after you received your invite (or if you were not invited how long after you became eligible) did you receive your first dose of vaccine?

☐ Less than 2 weeks *(Please go to question 12)*

☐ 2-3 weeks *(Please go to question 11)*

☐ 4 or more weeks *(Please go to question 11)*

☐ I had my vaccine before I was eligible *(Please go to question 12)*

11. Why were you not vaccinated sooner?

☐ I was not aware I was eligible

☐ No appointments available

☐ I preferred to wait to get vaccinated

☐ I delayed getting vaccinated because I had COVID-19

☐ I was isolating and did not wish to leave home to get vaccinated

☐ I did not have time

☐ Other ____

12. Please give the date of your first COVID-19 vaccine *It may be difficult to remember exactly; approximate dates are fine.*

D D / M M / Y Y

13. Please specify the brand/type of COVID-19 vaccine you had for your first dose?

☐ Pfizer

☐ AstraZeneca

☐ Unsure

14. How did you travel to the vaccination site?

☐ Walking/ cycling

☐ In a car alone or with members of own household

☐ In a car with member(s) from a different household

☐ Public transport

☐ Other ____

15. In the 3-4 weeks after receiving your first dose, how often have you met/mixed with others outside of your household (e.g. to go to shops, see friends and family)?

☐ I've mixed with people outside of my household for the same amount of time as I did before getting my vaccine

☐ I've mixed more with people outside of my household after getting the vaccine

☐ I've mixed less with people outside of my household after getting the vaccine

16. If you received a second dose, please give the date of your second COVID-19 vaccine *It may be difficult to remember exactly; approximate dates are fine.* D D / M M / Y Y

17. Please specify the brand/type of COVID-19 vaccine you had for your second dose?

☐ Pfizer

☐ AstraZeneca

☐ Unsure

18. How did you travel to the vaccination site for your second dose?

☐ Walking/ cyclingl

☐ In a car alone or with member(s) of own household

☐ In a car with member(s) from a different household

☐ Public transport

☐ Other ____

19. In the 3-4 weeks after receiving your second dose, how often have you met/mixed with others outside of your household (e.g. to go to shops, see friends and family)?

☐ I've mixed with people outside of my household for the same amount of time as I did before getting my vaccine

☐ I've mixed more with people outside of my household after getting the vaccine

☐ I've mixed less with people outside of my household after getting the vaccine

20. If you have not received a COVID-19 vaccine, please can you give us a reason from the options below

Please select all that apply

☐ I have not been called for a vaccine

☐ I was not aware I was eligible

☐ There were no appointments available

☐ I would prefer not to get vaccinated at the moment

☐ I expect to get vaccinated soon but have not had a vaccine yet

☐ I am delaying getting vaccinated because I have been unwell or have had COVID-19 infection…………………………………………………………………………………………………

……………………………………….

☐ I am isolating and do not wish to leave home to get vaccinated

☐ I have not had time

☐ Other

____

Part 3: Details about your illness before getting tested for COVID-19

21. Why were your tested for COVID-19?

☐ I had COVID-19 symptoms

☐ In contact with a case

☐ I was tested in a care home

☐ I was tested in hospital

☐ I was tested as part of surge testing for a variant in my area

☐ I had another illness

☐ Other____

When you were tested on <<testdate>>, our records showed that you had COVID-19 symptoms starting on <<symptomdate>>. Can you please provide further details about your symptoms.

22. Please confirm if you had symptoms

☐ Yes, I had symptoms *(Please go to question 23)*

☐ No, I did not have symptoms *(Please go to Part 4)*

23. If the date that your symptoms started (<<symptomdate>>) is incorrect, please update when you had your first symptoms.

D	D	/	M	M	/	Y	Y

24. Which of the following symptoms did you have?

Please tick all that apply

☐ Fever or chills

☐ Cough *(Please go to question 21)*

☐ Sore throat

☐ Nausea

☐ Headache

☐ Fatigue

☐ Runny nose

☐ Shortness of breath

☐ Loss of taste and/or smell

☐ Diarrhoea

☐ Muscle/ body pain

☐ Other, please describe: ____

25. How severe would you describe your symptoms?

☐ Mild

☐ Moderate

☐ Severe

26. Have you accessed any healthcare services during your illness (either in person or over the phone)?

Please tick all that apply

☐ GP *(Please complete Part 4)*

☐ NHS 111 *(Please complete Part 4)*

☐ A&E Department *(Please complete Part 4)*

☐ Hospital *(Please go to question 27)*

☐ None of the above *(Please complete Part 4)*

27. Did you get admitted to hospital due to your illness?

☐ Yes *(Please go to question 28)*

☐ No *(Please complete Part 4)*

28. If hospitalised, what was the reason for your hospital admission?

☐ COVID-19 related

☐ Unrelated to COVID-19

29. What was your date of admission to the hospital? *It may be difficult to remember exactly; approximate dates are fine.*

D	D	/	M	M	/	Y	Y

30. How many days were you in the hospital?

______________________________ days

31. Did you receive oxygen in hospital?

☐ Yes ☐ No ☐ Unsure

32. Were you admitted to ICU/ITU (intensive care)?

☐ Yes ☐ No ☐ Unsure

33. Did you receive care involving a ventilator?

☐ Yes ☐ No ☐ Unsure

Part 4: Details in the week before you had symptoms (or a test taken if you did not have symptoms)

34. In the week before you had symptoms (or your COVID-19 test if you did not have symptoms) were you in contact with someone who was unwell with COVID-19 symptoms?

☐ Yes ☐ No ☐ Unsure

35. In the week before you had symptoms (or your COVID-19 test if you did not have symptoms) were you in contact with someone who tested positive for COVID-19?

☐ Yes ☐ No ☐ Unsure

36. In the week before you had symptoms (or your COVID-19 test if you did not have symptoms), did anyone visit your home?

Please tick all that apply

☐ No

☐ Yes, a friend or relative

☐ Yes, a carer

☐ Yes, a doctor or nurse

☐ Another person please describe

____

37. In the week before you had symptoms (or your COVID-19 test if you did not have symptoms), did you go to a shop or supermarket?

☐ Yes ☐ No ☐ Unsure

38. In the week before you had symptoms (or your COVID-19 test if you did not have symptoms), did you travel in a car with someone outside your home?

☐ Yes ☐ No ☐ Unsure

39. In the week before you had symptoms (or your COVID-19 test if you did not have symptoms), did you go indoors somewhere not in your home where other people go as well (e.g. place of worship, workplace)?

☐ Yes ☐ No ☐ Unsure

40. In the week before you had symptoms (or your COVID-19 test if you did not have symptoms), did you seek medical care outside your home (e.g. dentist, GP, hospital)?

☐ Yes ☐ No ☐ Unsure

41. In the week before you had symptoms (or your COVID-19 test if you did not have symptoms), did you use public transport (e.g. bus, tube)?

☐ Yes ☐ No ☐ Unsure

42. Have you been vaccinated with this season's flu vaccine (since September 2020)?

☐ Yes ☐ No ☐ Unsure

43. Please add any additional information you think would be useful for us to know (e.g. I was part of a vaccine clinical trial)

____

____

____
