# Supplementary material for: Bias assessment of a test-negative design study of COVID-19 vaccine effectiveness used in national policymaking
Source: Nat Commun. 2023 Jul 6;14:3984. doi: 10.1038/s41467-023-39674-0 (PMC10325974; doi:10.1038/s41467-023-39674-0)
Supplement: Supplementary file 3 — Reporting Summary [file 41467_2023_39674_MOESM3_ESM.pdf]

nature portfolio

Corresponding author(s): Sophie Graham

Last updated by author(s): Jun 20, 2023

Reporting Summary

Nature Portfolio wishes to improve the reproducibility of the work that we publish. This form provides structure for consistency and transparency in reporting. For further information on Nature Portfolio policies, see our Editorial Policies and the Editorial Policy Checklist.

Statistics

For all statistical analyses, confirm that the following items are present in the figure legend, table legend, main text, or Methods section.

n/a	Confirmed	
☐	☒	The exact sample size (*n*) for each experimental group/condition, given as a discrete number and unit of measurement
☐	☒	A statement on whether measurements were taken from distinct samples or whether the same sample was measured repeatedly
☐	☒	The statistical test(s) used AND whether they are one- or two-sided<br>*Only common tests should be described solely by name; describe more complex techniques in the Methods section.*
☐	☒	A description of all covariates tested
☐	☒	A description of any assumptions or corrections, such as tests of normality and adjustment for multiple comparisons
☐	☒	A full description of the statistical parameters including central tendency (e.g. means) or other basic estimates (e.g. regression coefficient) AND variation (e.g. standard deviation) or associated estimates of uncertainty (e.g. confidence intervals)
☐	☒	For null hypothesis testing, the test statistic (e.g. *F*, *t*, *r*) with confidence intervals, effect sizes, degrees of freedom and *P* value noted<br>*Give P values as exact values whenever suitable.*
☒	☐	For Bayesian analysis, information on the choice of priors and Markov chain Monte Carlo settings
☒	☐	For hierarchical and complex designs, identification of the appropriate level for tests and full reporting of outcomes
☒	☐	Estimates of effect sizes (e.g. Cohen's *d*, Pearson's *r*), indicating how they were calculated

Our web collection on statistics for biologists contains articles on many of the points above.

Software and code

Policy information about availability of computer code

Data collection	The programming code for this project is available on Github: https://github.com/grahams99/Enhanced_surveillance_questionnaire.
Data analysis	Stata (version 17) and R (version 4.1.3).

For manuscripts utilizing custom algorithms or software that are central to the research but not yet described in published literature, software must be made available to editors and reviewers. We strongly encourage code deposition in a community repository (e.g. GitHub). See the Nature Portfolio guidelines for submitting code & software for further information.

Data

Policy information about availability of data

All manuscripts must include a data availability statement. This statement should provide the following information, where applicable:

- Accession codes, unique identifiers, or web links for publicly available datasets
- A description of any restrictions on data availability
- For clinical datasets or third party data, please ensure that the statement adheres to our policy

Access to pseudonymised national datasets used in this study (National Immunisation Management Service and Second Generation Surveillance System) is managed by NHS England through the NHS COVID-19 Data Store: https://www.england.nhs.uk/contact-us/privacy-notice/how-we-use-your-information/covid-19-response/nhs-covid-19-data-store/. Questionnaire data was collected for the purposes of public health service evaluation and consent was not obtained for further sharing for research. If individuals would like to request UKHSA data you then they can contact DataAccess@ukhsa.gov.uk.

Human research participants

Policy information about studies involving human research participants and Sex and Gender in Research.

Reporting on sex and gender	Results are reported by and adjusted for sex.
Population characteristics	The first published study included individuals in England aged ≥70 years who had a COVID-19 test in the community with self-reported symptoms and a symptom onset date between 8th December 2020 and 21st February 20214. Patients were excluded if they had a history of a previous positive COVID-19 test from 26th October 2020 until 7th December 2020. The current study included individuals with a test from 1-21 February 2021 that responded a questionnaire that asked these individuals about their risk behaviors,
Recruitment	All individuals that met the study selection criteria were recruited into the study. Questionnaires were sent by post and also using a snap survey link via email address. Only those that responded to the questionnaire were included in the final study population. The questionnaire could have introduced some selection bias as individuals that responded to the questionnaire could have had better health-seeking behaviours. However, this was one of the objectives of the study. We did not find evidence of selection bias since, although the popualtion characteristics differed between respondents versus non-respondents of the questionnaire, the main study outcome (vaccine effectiveness) did not differ between these samples.
Ethics oversight	There was no ethics required since this was conducted as part of public health services evaluation.

Note that full information on the approval of the study protocol must also be provided in the manuscript.

Field-specific reporting

Please select the one below that is the best fit for your research. If you are not sure, read the appropriate sections before making your selection.

☒ Life sciences ☐ Behavioural & social sciences ☐ Ecological, evolutionary & environmental sciences

For a reference copy of the document with all sections, see nature.com/documents/nr-reporting-summary-flat.pdf

Life sciences study design

All studies must disclose on these points even when the disclosure is negative.

Sample size	23,713 individuals were sent the questionnaire. 8,649 responded and therefore made up the final study population. We did not do a formal power calculation since the objectives of the study were mainly descriptive and therefore all individuals that met the study inclusion criteria and none of the exclusion criteria were included.
Data exclusions	Individuals that did not respond to the questionnaire, which was 15,065 individuals.
Replication	The exact questionnaire is within the supplement and the programming code has been added to Github in this location: https://github.com/grahams99/Enhanced_surveillance_questionnaire.
Randomization	Not relevant since this is an observational study.
Blinding	Not relevant since this is an observational study.

Reporting for specific materials, systems and methods

We require information from authors about some types of materials, experimental systems and methods used in many studies. Here, indicate whether each material, system or method listed is relevant to your study. If you are not sure if a list item applies to your research, read the appropriate section before selecting a response.

Materials & experimental systems

n/a	Involved in the study	
☒	☐	Antibodies
☒	☐	Eukaryotic cell lines
☒	☐	Palaeontology and archaeology
☒	☐	Animals and other organisms
☒	☐	Clinical data
☒	☐	Dual use research of concern

Methods

n/a	Involved in the study	
☒	☐	ChIP-seq
☒	☐	Flow cytometry
☒	☐	MRI-based neuroimaging
